# Robotic versus laparoscopic proctectomy: a comparative study of short-term economic and clinical outcomes

**DOI:** 10.1007/s00384-023-04446-1

**Published:** 2023-06-07

**Authors:** José Tomás Larach, Julie Flynn, Michelle Tew, Diharah Fernando, Sameer Apte, Helen Mohan, Joseph Kong, Jacob J. McCormick, Satish K. Warrier, Alexander G. Heriot

**Affiliations:** 1 Division of Cancer Surgery, Peter MacCallum Cancer Centre, Victorian Comprehensive Cancer Centre, Melbourne, Australia; 2 Department of Oncology, Sir Peter MacCallum Cancer Centre, University of Melbourne, Melbourne, Australia; 3 Department of Digestive Surgery, Pontificia Universidad Católica de Chile, Santiago, Chile; 4 General Surgery and Gastrointestinal Clinical Institute, Epworth Healthcare, Melbourne, Australia; 5 Health Economics, Department of Health Services Research, Peter MacCallum Cancer Centre, Victorian Comprehensive Cancer Centre, Melbourne, Australia; 6 Central Clinical School, Monash University, Melbourne, Australia

**Keywords:** Robotic, Laparoscopic, Colorectal, Proctectomy, Costs, Economic outcomes

## Abstract

**Background:**

Although several studies compare the clinical outcomes and costs of laparoscopic and robotic proctectomy, most of them reflect the outcomes of the utilisation of older generation robotic platforms. The aim of this study is to compare the financial and clinical outcomes of robotic and laparoscopic proctectomy within a public healthcare system, utilising a multi-quadrant platform.

**Methods:**

Consecutive patients undergoing laparoscopic and robotic proctectomy between January 2017 and June 2020 in a public quaternary centre were included. Demographic characteristics, baseline clinical, tumour and operative variables, perioperative, histopathological outcomes and costs were compared between the laparoscopic and robotic groups. Simple linear regression and generalised linear model analyses with gamma distribution and log-link function were used to determine the impact of the surgical approach on overall costs.

**Results:**

During the study period, 113 patients underwent minimally invasive proctectomy. Of these, 81 (71.7%) underwent a robotic proctectomy. A robotic approach was associated with a lower conversion rate (2.5% versus 21.8%;P = 0.002) at the expense of longer operating times (284 ± 83.4 versus 243 ± 89.8 min;P = 0.025). Regarding financial outcomes, robotic surgery was associated with increased theatre costs (A$23,019 ± 8235 versus A$15,525 ± 6382; P < 0.001) and overall costs (A$34,350 ± 14,770 versus A$26,083 ± 12,647; P = 0.003). Hospitalisation costs were similar between both approaches. An ASA ≥ 3, non-metastatic disease, low rectal cancer, neoadjuvant therapy, non-restorative resection, extended resection, and a robotic approach were identified as drivers of overall costs in the univariate analysis. However, after performing a multivariate analysis, a robotic approach was not identified as an independent driver of overall costs during the inpatient episode (P = 0.1).

**Conclusion:**

Robotic proctectomy was associated with increased theatre costs but not with increased overall inpatient costs within a public healthcare setting. Conversion was less common for robotic proctectomy at the expense of increased operating time. Larger studies will be needed to confirm these findings and examine the cost-effectiveness of robotic proctectomy to further justify its penetration in the public healthcare system.

**Supplementary Information:**

The online version contains supplementary material available at 10.1007/s00384-023-04446-1.

## Introduction

The worldwide adoption of minimally invasive colorectal surgery over the last decades has been remarkable but it is still limited [1, 2]. Specifically for laparoscopic proctectomy, randomised controlled trials support an improved recovery and equivalent long-term oncologic outcomes compared to an open approach [3–6]. Furthermore, despite increased operative costs, it is well accepted that laparoscopic surgery is cost-effective compared to open colorectal surgery, which is mainly explained by lower complication rates and a shorter length of hospital stay [7–9]. Undertaking a laparoscopic approach to proctectomy, however, is far from easy. Even in the hands of experienced minimally invasive surgeons, high conversion rates translate the challenges of such an approach [10].

Robotic surgery offers advantages over conventional laparoscopic surgery, including the ability to rotate and articulate instruments, a surgeon-controlled camera with three-dimensional vision, and improved ergonomics. However, one disadvantage of robotic surgery is the lack of tactile feedback. Despite this limitation, several randomized controlled trials have demonstrated that robotic proctectomy is not only feasible and safe, but also associated with lower conversion rates compared to laparoscopic proctectomy, albeit with longer operating times [11, 12]. Nevertheless, long-term oncologic outcomes and cost data will be required before justifying further penetration of robotic colorectal surgery.

The costs of robotic colorectal surgery are perceived to be substantially increased compared to conventional laparoscopic surgery [13–15]. Although several studies in the literature compare the clinical outcomes and costs of laparoscopic and robotic proctectomy, most of them reflect outcomes following the utilisation of single-quadrant, older generation platforms, performing hybrid procedures [16–19]. The aim of this study is to compare the short-term financial and clinical outcomes of robotic and laparoscopic proctectomy within a public healthcare system, utilising a multi-quadrant platform, beyond the institutions’ learning curve for robotic colorectal surgery.

## Materials and methods

### Study design

A retrospective review of a prospectively maintained rectal cancer databases in a public quaternary centre (Peter MacCallum Cancer Centre, Melbourne, Australia) was carried out. Consecutive patients undergoing minimally invasive (laparoscopic and robotic) proctectomy between January 2017 and June 2020 were included. Patients who had resections due to lesions higher than 15 cm from the anal verge were excluded. Demographic characteristics, baseline clinical, tumour and operative variables, perioperative, histopathological outcomes and costs were compared between the laparoscopic and robotic groups. Subsequently, univariate, and multivariate analyses utilising demographic, baseline clinical and surgical variables were performed to identify the drivers of costs during the inpatient episode and examine the impact of the surgical approach on the overall costs.

### Definitions and clinical outcomes

All cancer cases were discussed in a multidisciplinary team meeting. The surgical approach was decided on a case-by-case basis with input from a multidisciplinary team discussion and the robotic platform availability.

The clinical and histopathological staging was recorded according to the TNM classification (AJCC 8^th^ Edition for Cancer Staging) [20]. The pre-treatment imaging assessment and re-staging were based on results from a CT, PET-CT, and pelvic MRI. Tumour height was defined clinically, endoscopically or by the pre-treatment MRI, with the anal verge as the reference point.

Extended resections were defined as per the Beyond TME Collaborative as any procedure that required an *en-bloc* removal of an adjacent pelvic organ due to invasion or circumferential margin threatening by the primary rectal tumour [21]. Pelvic sidewall dissections were indicated when there were suspicious lymph nodes in the common or external iliac territories, obturator fossa or internal iliac system based on size criteria in the MRI (short-axis equal or greater than 7 mm in the pre-treatment MRI, and equal or greater than 5 mm in the post-treatment MRI if long-course chemoradiation therapy was delivered).

Complications, readmissions, and mortality up to 90 days after surgery were considered and recorded. Complications were classified according to the Clavien-Dindo classification [22].

The histopathological evaluation considered an R0 resection as a resection margin of > 1 mm. R1 resection was the presence of microscopic residual disease 1 mm or less from the resection margin, whereas R2 resection was the presence of macroscopic residual disease.

### Financial outcomes

Individual patients’ costs were retrieved via the Business Intelligence and Analytics Department of the hospital. These included real costs from the inpatient episode, thus no estimated values were used. All costs were recorded as Australian Dollars (A$) and were obtained from the databases in November 2021. Costs were classified in theatre, hospitalisation, and overall costs. Theatre costs included consumables, medications, nursing staff, theatre utilisation and recovery expenses. Hospitalisation costs included ICU and ward expenses, medical and nursing staff, allied health staff, medication, transfusions and laboratory and imaging testing costs. Overall costs were the combination of total theatre and hospitalisation costs.

### Procedures

In our institution, a robotic colorectal surgery program was started in 2010 and taTME started in 2015. More than 200 robotic cases and more than 100 taTME procedures were performed by 4 and 2 surgeons, respectively, before the period of this study. All surgeons were formally trained in colorectal surgery and beyond the learning curve for laparoscopic TME. All surgeons were robotically credentialed by the industry (Intuitive Surgical). All patients had mechanical bowel preparation. General anaesthetic was given, and prophylactic antibiotics were administered at induction. An indwelling urinary catheter was inserted, and the patient was placed in the Lloyd-Davies position. All patients had sequential compression devices and low molecular weight heparin was given on induction, and throughout the hospital stay. The splenic flexure was mobilised in all restorative cases and selectively in non-restorative cases. For cancer patients, a high ligation of the inferior mesenteric vessels was performed. The approach was selected by the treating surgeon based on patient and team factors, plus the availability of the robotic platform. Laparoscopic TME was undertaken utilising a five-trocar technique. The dissection followed the TME plane which was continued down to the level of the pelvic floor and then, the rectal transection was carried out using laparoscopic linear staplers in restorative cases. All robotic TME cases were performed with the Da Vinci Xi Surgical System (Intuitive Surgical, Sunnyvale, CA, USA). Four robotic ports and an assistant port were used in all cases. Procedures were performed in a totally robotic fashion. After the pelvic dissection, rectal transection was carried out utilising robotic linear staplers if a restorative case was performed. When a taTME approach was added, the procedure was carried out as a synchronous two-team operation. The transabdominal component of taTME was either robotic or laparoscopic according to the surgeons’ preference and platform availability. Details of our setup and steps have been previously published by the authors [23]. Anastomoses in the taTME group were mostly performed utilising a single-stapled double-purse string technique as described in the literature [24].

All patients followed an ERAS protocol perioperatively.

### Statistical analysis

Continuous variables were described as mean and SD. Categorical variables were described as frequencies and percentages. Chi-square or Fisher’s exact tests were utilised to compare categorical variables. Students’ t-test was used to compare continuous variables. Given the skewed distributions of economic outcomes, a GLM with gamma distribution and log-link function was used to identify the drivers of overall costs in this study. Those variables with a P value < 0.1 in the simple linear regression analysis were considered significant and were entered into the GLM. Additionally, a GLM in a backwards fashion was conducted by manually removing variables starting from the one with the highest P-value until only keeping those significantly related to overall costs. All statistical analyses were performed using the IBM SPSS Statistics for Windows, Version 27.0 (Armonk, NY, IBM Corp).

### Ethics

A local Institutional Review Board ethics approval was obtained (QA/80014/PMCC-2021-283105).

## Results

One hundred and thirteen patients underwent MIS proctectomy during the study period. Of these, 81 (71.7%) underwent a robotic proctectomy. In terms of operation variables, a taTME component was more often utilised in the laparoscopic approach (34.4% versus 13.6%; P = 0.003). Two patients had a re-do proctectomy in the robotic group. The rest of the patients had a primary rectal resection. The complete list of demographics, clinical and tumour baseline variables and operative variables are summarised in Table 1. The details of the extended resections performed are shown in the Supplementary Table 1.

**Table 1:** Demographic and baseline clinical and operative variables

**Variable**	**Laparoscopic (n = 32)**	**Robotic (n = 81)**	**p**
**Age in years, mean (SD)**	58.43 (14)	60 (13.7)	0.35
**Age > 70 years, n (%)**	6 (18.8)	25 (30.9)	0.24
**Sex, n (%)**			0.11
Male	18 (56.3)	59 (72.8)	
Female	14 (43.7)	22 (27.2)	
**BMI kg/m**^**2**^**, mean (SD)**	27.4 (6.7)	28 (6.1)	0.99
**BMI ≥ 30 kg/m**^**2**^	10 (31.3)	19 (23.5)	0.47
**ASA, n (%)**			0.47
I-II	24 (75)	60 (74.1)	
III-IV	5 (15.6)	21 (25.9)	
Missing	3 (9.4)	0 (0)	
**Indication, n (%)**			0.55
Cancer	32 (100)	78 (96.3)	
Benign pathology	0 (0)	3 (3.7)	
**Distance from anal verge, mean (SD)**	8.68 (4.71)	8 (3.71)	0.68
**Tumour location, n (%)**			1
Lower rectum	9 (28.1)	21 (25.9)	
Mid rectum	23 (71.9)	55 (67.9)	
**Clinical T, n (%)**			0.14
1–2	5 (15.6)	24 (29.6)	
3–4	23 (71.9)	48 (59.3)	
Missing	4 (12.5)	4 (4.9)	
**Clinical N, n (%)**			1
-	14 (43.8)	36 (44.4)	
+	14 (43.8)	35 (43.2)	
Missing	4 (12.5)	4 (4.9)	
**Metastatic disease on diagnosis**	6 (18.8)	1 (1.2)	0.00
**Neoadjuvant therapy, n (%)**	23 (71.9)	49 (60.5)	0.5
**Procedure, n (%)**			0.39
Restorative	25 (78.1)	70 (86.4)	
Non-restorative	7 (21.9)	11 (13.6)	
**Stoma type, n(%)**			0.42
** No stoma**	1 (3.1)	6 (7.4)	
** Loop ileostomy**	24 (75)	64 (79)	
** End colostomy**	7 (21.9)	11 (13.5)	
**taTME approach, n (%)**	11 (34.4)	11 (13.6)	0.00
**Extended resection, n (%)**	5 (15.6)	10 (12.3)	0.75
**Pelvic sidewall lymph node dissection, n (%)**	2 (6.3)	5 (6.2)	1

*SD* Standard deviation, *ASA* American Society of Anaesthesiologists Classification, *BMI* body mass index, *taTME* transanal total mesorectal excision

Regarding perioperative outcomes, a robotic approach was associated with a lower conversion rate (2.5% versus 21.8%; P = 0.002) and longer operating times (284 ± 83.4 versus 243 ± 89.8 minutes; P = 0.025). Causes for conversion in the robotic group were a proximal IMA lesion (n = 1), obesity (n = 1), and intraoperative bleeding (n =1). In the laparoscopic group the reasons for conversion included a difficult pelvic dissection (n = 4), inadequate bowel perfusion (n = 1), an anterior tumour perforation (n = 1) and an iliac artery lesion which was controlled with only 50 ml of bleeding (n = 1). Complications, reoperations, length of stay, readmissions, and surrogate oncologic markers were similar between groups (Table 2). It must be noted a non-significant difference between Clavien-Dindo III and IV complications amongst groups. The laparoscopic group had one reoperation due to bleeding (unidentified source intraoperatively). The robotic group had two small bowel obstructions due to paraostomal hernias, a high output ileostomy with subsequent prolapse, a small bowel obstruction in a trocar site, five pelvic collections or anastomotic leaks which required either a CT-drainage or a flexible sigmoidoscopy washout plus antibiotics, an anastomotic bleeding, and a pelvic bleeding with a perineal wound breakdown following an APR.

Regarding financial outcomes, robotic surgery was associated with increased theatre costs (A$23,019 ± 8235 versus A$15,525 ± 6382; P < 0.001) and overall costs (A$34,350 ± 14,770 versus A$26,083 ± 12,647; P = 0.003). Hospitalisation costs were similar between both approaches (Table 3).

**Table 2:** Perioperative outcomes

**Variable**	**Laparoscopic (n = 32)**	**Robotic (n = 81)**	**P**
**Conversion, n (%)**	7 (21.8)	2 (2.5)	0.00
**Operating time (minutes)**	243 (83.4)	284 (89.8)	0.02
**Length of hospital stay, days (SD)**	10 (5.1)	11 (7.7)	0.88
**Overall complications, n (%)**	13 (40.6)	42 (51.9)	0.30
**Clavien-Dindo, n (%)**			0.25
I-II	12 (37.5)	31 (38.3)	
III-IV	1 (3.1)	11 (13.5)	
**Reoperation, n (%)**	1 (3.1)	7 (8.6)	0.43
**30-day readmission, n (%)**	1 (3.1)	8 (9.9)	0.44
**Lymph nodes harvested (SD)**	17.6(6.4)	18 (7.1)	0.59
**R1 resection, n (%)**	3 (9.4)	4 (4.9)	0.40

*SD* Standard deviation

**Table 3:** Financial outcomes

**Variable**	**Laparoscopic (n = 32)**	**Robotic (n = 81)**	**p**
**Theatre costs, A$ (SD)**	15525 (6382)	23019 (8235)	0.00
**Hospitalisation costs, A$ (SD)**	10558 (9513)	11331 (10070)	0.36
**Total hospital costs, A$ (SD)**	26083 (12647)	34350 (14770)	0.00

*A$* Australian Dollars, *SD* Standard deviation

An ASA ≥ 3, non-metastatic disease, low rectal cancer, neoadjuvant therapy, non-restorative resection, extended resection, and a robotic approach were identified as drivers of overall costs in the simple linear regression analysis (P < 0.1). However, after performing a GLM including these factors, a robotic approach was not identified as an independent driver of overall costs during the inpatient episode (Table 4). When adjusted by complications, the results did not differ from the original analysis.

**Table 4:** Generalised linear model for baseline drivers of overall costs during the inpatient episode

Variable*	**Parameter**			
	**B**	**Standard Error**	**95% Confidence Interval**	**P value**
**Intercept**	10153	0.13	9909–6615	0.00
**ASA ≥ 3**	0.38	0.09	0.2–0.56	0.00
**Low rectal cancer**	0.02	0.08	-0.13–0.18	0.79
**Stage IV disease**	-0.39	0.18	-0.74–0.31	0.03
**Robotic approach**	0.14	0.09	0.31–2.71	0.1
**Restorative resection**	-0.04	0.11	-0.22–0.21	0.97
**Extended resection**	0.44	0.10	0.24–0.64	0.00

*ASA* American Society of Anaesthesiologists Classification

*Variables with a P value < 0.1 in the simple linear regression analysis were included in the model

An ASA ≥ 3, non-metastatic disease, and extended resections were significantly associated with increased overall costs in the multivariate analysis. Moreover, similar results were obtained after running the GLM in a backwards fashion and when adjusting for postoperative complications.

## Discussion

The current study shows that a robotic approach is associated with a decreased conversion rate at the expense of a longer operating time compared to laparoscopic proctectomy. Moreover, despite theatre costs being higher for robotic proctectomy, overall inpatient costs seem to be similar to those of laparoscopic proctectomy when adjusted for confounders.

Although robotic surgery is generally perceived as being more expensive than a conventional laparoscopic proctectomy, it could be said that the specialised literature is at least conflicting about the cost-benefit and cost-effectiveness outcome of robotic proctectomy. Several studies in the literature compare clinical outcomes and costs of laparoscopic and robotic proctectomy [16–19]. Most of these are retrospective or matched cohort studies showing that robotic proctectomy is associated with increased overall expenses, specially at the expense of theatre costs. All of these except one study, however, communicate outcomes of robotic cases performed with the S or Si Da Vinci platforms including hybrid procedures. Moreover, control for confounders is not necessarily present in the analyses, and most of them do not include sufficient data to perform formal cost-effectiveness analyses. Hence, the generalisation of these results should be seen with caution.

Only a few studies analyse the cost-effectiveness of robotic colorectal surgery. Simianu et al. [9] compared the cost-effectiveness of open, laparoscopic, and robotic proctectomy by performing a decision tree modelling analysis. They showed that open proctectomy had increased costs and lower QoL compared to minimally invasive approaches. From a societal perspective, a robotic proctectomy was $497 more expensive per case more than the laparoscopic approach, with minimal QoL improvements, resulting in an incremental cost-effectiveness ratio of $751,056 per QALY. From a healthcare perspective, a robotic approach was $983 more expensive per case with an incremental cost-effectiveness ratio of $1,485,139/QALY. In a probabilistic sensitivity analysis, the cost-effective approach to proctectomy was laparoscopic in 42% of cases, robotic in 39%, and open in 19% at a WTP of $100,000/QALY. A sensitivity analyses demonstrated that operative cost and length of stay were factors influencing cost-effectiveness, thus, the authors state that by achieving certain postoperative and cost thresholds, robotic surgery may become cost-effective. Nevertheless, several assumptions and the utilisation of empirical data and estimates in the model impede the generalisation of these conclusions.

As opposed to the previously mentioned studies, Quijano et al. [18] in 2020 performed a prospective study comparing the cost-effectiveness of laparoscopic and robotic rectal cancer surgery. Some 104 patients underwent laparoscopic surgery, whilst 81 patients had a robotic resection utilising the Si and Xi platforms. Theatre costs were significantly increased in the robotic group (4307€ versus 3835€; P = 0.04). Nevertheless, total inpatient costs were similar (7272.03€ for the robotic group and 6968.63€ for the laparoscopic group; P = 0.44). The mean QALY at 1 year for the robotic group was 0.8482, which was higher than that associated with laparoscopic surgery (0.6532) (P = 0.018). Probabilistic sensitivity analysis, estimated by Monte Carlo simulations at a WTP threshold of 20,000€ and 30,000€ showed that there was a 95.54% and 97.18% probability, that a robotic approach for proctectomy was more cost-effective than laparoscopic surgery. Considering these results, the authors concluded that a robotic proctectomy was more cost-effective than laparoscopic proctectomy.

Explanations to this dissonance may be related to the different platforms utilised and institutional and individual surgeons’ learning curves. Newer platforms allow for an easier delivery of totally robotic procedures as opposed to hybrid approaches which require the concomitant use of laparoscopic and robotic equipment. In this line, recent studies show that the evolution of robotic platforms is associated with an increasing case volume and reduced operating times and costs within this field [25–27]. It must also be considered that the robotic surgery market has been driven by one main provider during the last two decades, and that the arrival of competitors should allow for costs to be diminished and favour the evolution of platforms towards incremental versatility. In an analogous scenario, and non-surprisingly in hindsight, Aly et al. [8] showed that laparoscopic colorectal surgery is becoming more cost-effective over time. In this systematic review, the authors showed that the cost-difference percentage between both approaches is decreasing over time (R-value =  − 0.44; P = 0.046), projecting that the results of future economic evaluations would unequivocally show that laparoscopic surgery is cheaper than open surgery. The initial uptake of laparoscopic colorectal surgery was associated with increased theatre costs and similar overall costs [28]. However, as surgeons move beyond the learning curves, the approach is normalised within healthcare institutions, the equipment costs decrease due to competition, and clinical benefits are maximised with the subsequent reduce in overall costs [29].

This study has several limitations to be addressed. As a retrospective cohort study, selection biases cannot be excluded, and the small number of patients make it subject to statistical type II errors. The fact of having a high rate of an added taTME component may also bias our results. However, our data shows that the addition of a taTME component to either a laparoscopic or a robotic approach was not associated with higher overall costs ($A 28724 versus $A 26130, p = 0.54706 and $A 34587 versus $A 43730; p = 0.034, respectively). The lower costs associated with taTME in the robotic group may be due to a statistical error secondary to the limited sample size (11 of 81 cases). Furthermore, indirect costs incurred due to losses such as lost wages, productivity, and costs resulting from the need for home care and others, were not measured in this case. Also, despite theatre costs are considered, the capital expenses of running and maintaining a robotic and laparoscopic surgery program are not evaluated in this study. Moreover, this work does not include the necessary variables to deliver a cost-effectiveness analysis which would be the ideal template to compare both approaches. The ideal setting to give an answer to this matter would be to perform a randomised controlled trial with costing and quality of life outcomes. Nevertheless, to our best knowledge, this is the first study comparing the costs of minimally invasive proctectomy within a public Australian healthcare system and provides an insight to potential areas of cost reduction to maximise the cost-benefit ratio of the approach.

Robotic colorectal surgery is still in the early adopters’ phase where institutions are negotiating their learning curves. Regardless, limited trials show that robotic proctectomy is associated with an improved bowel recovery and lower conversion rates, at the expense of increased operating time [11]. Additionally, recent research also demonstrates that robotic proctectomy may be associated with enhanced functional outcomes over laparoscopic proctectomy [30, 31]. Moving beyond learning curves and an increased market competition with the arrival of different platforms may dramatically change our current perception of the utility and cost-benefit and cost-effectiveness of robotic colorectal surgery in years to come.

## Conclusion

Robotic proctectomy is associated with increased theatre costs but not with increased overall inpatient costs within a public healthcare setting. Conversion was less common for robotic proctectomy at the expense of increased operating time. Larger studies will be needed to confirm these findings and examine the cost-effectiveness of robotic proctectomy.

### Supplementary Information

Below is the link to the electronic supplementary material.

Supplementary file1 (DOCX 14 KB)

## Data Availability

Data is available upon reasonable request to the Editor.
