# Supplementary material for: Robotic versus laparoscopic proctectomy: a comparative study of short-term economic and clinical outcomes
Source: Int J Colorectal Dis. 2023 Jun 7;38(1):161. doi: 10.1007/s00384-023-04446-1 (PMC10247549; doi:10.1007/s00384-023-04446-1)
Supplement: Supplementary file 1 — Supplementary file1 (DOCX 14 KB) [file 384_2023_4446_MOESM1_ESM.docx]

**Supplementary Table 1. Details of the extended resections performed in this series**

| Laparoscopic taTME + posterior bladder wall cuff + seminal vesicles + right pelvic side-wall + loop ileostomy |
| --- |
| Laparoscopic APR + left ovarian cystectomy + right pelvic sidewall dissection (converted) |
| Laparoscopic taTME + presacral fascia + left pevic side-wall + intraoperative radiotherapy |
| Laparoscopic APR + presacral fascia + coccyx |
| Laparoscopic APR + sacrectomy |
| Robotic Redo-ULAR + en bloc radical prostatectomy for resection of rectourethral cutaneous fistula |
| Robotic, converted to open ULAR/taTME + right pelvic sidewall + repair aortic injury + repair right external iliac vein + partial vaginectomy + right ureterolysis + loop ileostomy |
| Robotic APR + en bloc radical prostatectomy |
| Robotic ULAR + taTME + hysterectomy and posterior vaginectomy + left pelvic sidewall + loop ileostomy |
| Robotic pelvic exenteration with taTME and en-bloc cysto-prostatectomy, bilateral ureterolysis, small bowel resection & anastomosis, parastomal hernia repair, formation of ileal conduit & loop ileostomy |
| Robotic ULAR + en bloc cystoprostatectomy + ileal conduit |
| Robotic ULAR + en bloc hysterectomy and bilateral salpingo-oophorectomy with partial peritonectomy + bilateral ureterolysis + loop ileostomy |
| Robotic Redo-ULAR + small bowel resection + right hemicolectomy + right seminal vesicle + loop ileostomy |
| Robotic APR + hysterectomy + posterior vaginectomy + IGAM flap |
| Robotic ULAR + right seminal vesicle + loop ileostomy |
